# Tunable Lifetime and Nonlinearity in Two Dimensional Materials Plasmonic-Photonic Absorber

**DOI:** 10.3390/nano12030416

**Published:** 2022-01-27

**Authors:** Renlong Zhou, Sa Yang, Yongming Zhao

**Affiliations:** School of Physics and Information Engineering, Guangdong University of Education, No. 351 Xinggang Road, Guangzhou 510303, China; yangsa@gdei.edu.cn (S.Y.); zhaoym@gdei.edu.cn (Y.Z.)

**Keywords:** plasmonic-photonic absorber, lifetime, nonlinearity

## Abstract

We investigate a framework of local field, quality factor and lifetime for tunable graphene nanoribbon plasmonic-photonic absorbers and study the second order and third order nonlinear optical response of surface plasmons. The energy exchange of plasmonic-photonic absorber occurs in two main ways: one way is the decay process of intrinsic loss for each resonant mode and another is the decay process of energy loss between graphene surface plasmon (GSP) mode and the external light field. The quality factor and lifetime of the plasmonic-photonic absorber can be obtained with using the coupled mode theory (CMT) and finite difference time domain (FDTD) method, which are effectively tunable with changing Fermi energy, carrier mobility and superstrate refractive index. The evolutions of total energy and lifetime of GSP are also shown, which are helpful for the study of micro processes in a two-dimensional material plasmonic-photonic absorber. The strongly localized fundamental field induces a desired increase of second harmonic (SH) wave and third harmonic (TH) wave. The manipulation of the quality factor and lifetime of the GSP makes graphene an excellent platform for tunable two-dimensional material plasmonic-photonic devices to realize the active control of the photoelectric/photothermal energy conversion process and higher harmonic generation.

## 1. Introduction

The two-dimensional (2D) materials, such as graphene and black phosphorus, have fantastic and unique properties, such as being dynamically tunable with chemical doping or electrostatic gate, which makes 2D materials an excellent plasmonic platform for dynamically tunable devices [1,2,3,4]. Compared to metal, the improved absorption and confinement of surface plasmon (SP) in graphene-like 2D materials attracts a promising interest to realize nanoscale integrated photonic and electronic circuits due to their ability for controlling and confining waves at the subwavelength scale [5,6,7]. To controlling and confining waves in graphene, graphene can be incorporated into optical cavities, dielectric gratings, and photonic crystals. In addition, light can be efficiently trapped and absorbed. It has been shown that the structured graphene derivatives yield remarkably high optical absorption, even though a single atomic sheet of graphene can absorb only 2.3% of light in the infrared to visible spectral. As a relatively new novel optical function material, 2D materials such as graphene photonic devices are realized, such as light absorption, slow light, mode-locking, etc. Moreover, the graphene can support the generation and propagation of SP. Binary graphene nanoribbons have been theoretically proposed. GSP has the opposite in-plane electron oscillations along its two surfaces. In recent experiments, the tunability of doped SP in mid-infrared frequency has been experimentally demonstrated and theoretically studied [8,9,10,11]. The Fermi energy has a value of 1.17 eV. The carrier mobility ranges from ~1000 cm^2^/(V·s) in chemical vapor deposition grown graphene to 230,000 cm^2^/(V·s) in suspended exfoliated graphene. The plasmonic-photonic absorber and their ultrafast dynamics in the photoelectric/photothermal conversion device have broad application fields of photonics, photodetectors [12,13,14] and biomedicine [15,16]. The design and control of SP-induced hot electrons is proposed, which is based on the heterojunction characteristics [17,18,19,20]. There are many advantages of SP-induced hot electrons. The rapid transfer and collection (less than 100 fs) of hot electrons can avoid the energy loss and extension of response time caused by relaxation, recombination, binding and other processes. After the hot electrons enter the 2D materials through the heterojunction interface, the enhanced photoelectric/photothermal conversion efficiency is obtained. On the other hand, the strongly localized fundamental field of SP induces a desired increase of second harmonic wave and third harmonic wave [21,22]. The high-efficiency second and third harmonic generation effects have been studied in monolayer graphene-based transistors; they have also been experimentally observed in single-layer and bi-layer graphene sheets [22]. For a free-standing graphene, the second-order nonlinearity is forbidden due to the centrosymmetric of its structure [23,24]. Despite its center symmetry, the symmetry breaking can induce the second order nonlinearity in graphene [25,26,27,28]. The coupled graphene-cavity system was described by using coupled mode theory (CMT) [29]. The surface second harmonic generation in the graphene/vicinal-SiC structure is observed with large second-order susceptibility (1.99 × 10^−10^ m/V) [30].

The ultrafast dynamics of excited carriers in graphene was experiment observed, which can disentangle the subsequent decay into excitations of acoustic phonons and optical phonons [31]. In this work, we set a framework to study the characteristic of graphene nanoribbons (GN) plasmonic-photonic absorbers, including the local field, density of photon flux, quality factor and lifetime, and to study the second order and third-order nonlinear optical response of GSP. A theoretical model of this framework is established using the FDTD simulation and CMT analysis [32,33]. The nanostructured GN grating structure can enhance the light-matter interaction and plasmonic-photonic absorption. We have studied wave confinement, photon flux density, quality factor and lifetime of tunable surface plasmon in subwavelength scales. The quality factor and lifetime for each process of intrinsic loss and coupling loss have been especially studied. We describe the dual graphene-cavity model with a CMT analysis. The absorption can vary with Fermi energy, carrier mobility and refractive index of superstrate, which are calculated by FDTD. The results obtained with the FDTD method agree well with the results of CMT analysis. The decay rate, quality factor and lifetime in CMT are obtained from theoretical fitting of exact values with FDTD simulation. The evolutions of total energy and lifetime of GSP modes are also shown for short pulse, which are helpful for study of micro process in graphene plasmonic-photonic absorber. The strongly localized fundamental field induces a desired increase of TH wave and SH wave, which includes second harmonic signal, sum frequency signal and difference frequency signal in the SH wave. These methods are useful for investigating the optical intrinsic loss process or optical coupling loss process in 2D materials based plasmonic-photonic devices to realize the active control of the photoelectric/photothermal energy conversion process such as solar energy conversion [34], photoelectric conversion [35], nanoantenna [36], plasmonic hot carriers controlled higher harmonic generation [37] and high-sensitivity sensing [38,39]. The ability to tune the maximal radiative quality factor from infinite to finite is a unique property for trapped light within the radiation continuum.

## 2. Nanostructured GN and Theoretical Analysis

**Figure 1:**
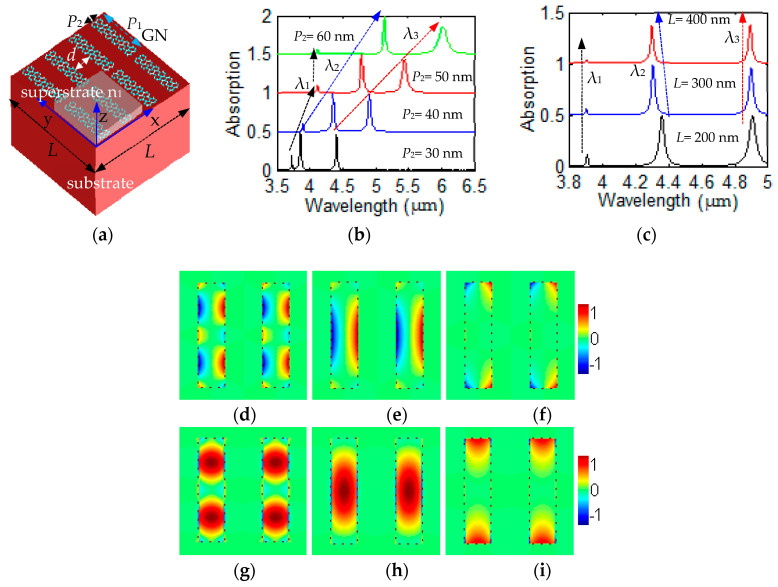

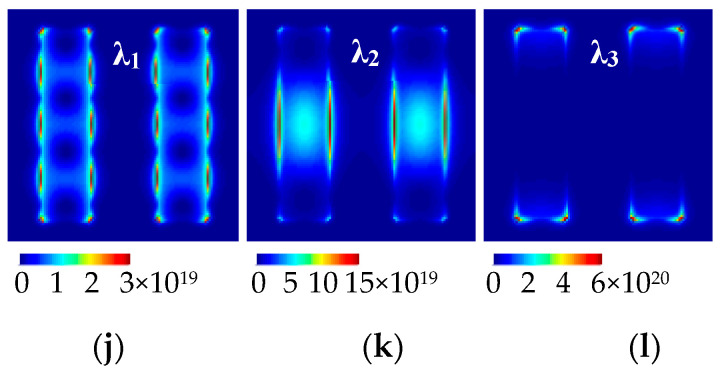
(**a**) The nanostructured GN surrounded with substrate and superstrate. (**b**) The absorption with different width *P*_2_ = 30 nm, 40 nm, 50 nm and 60 nm, respectively. (**c**) The absorption with different lattice period *L* = 200 nm, 300 nm and 400 nm, respectively. The distributions of electric field **E**_z_ at (**d**) λ_1_ = 3.9 μm, (**e**) λ_2_ = 4.35 μm, and (**f**) λ_3_ = 4.90 μm, respectively. The distributions of electric field **E**_x_ at (**g**) λ_1_ = 3.9 μm, (**h**) λ_2_ = 4.35 μm, and (**i**) λ_3_ = 4.90 μm, respectively. The distributions of photon flux density Φ at (**j**) λ_1_ = 3.9 μm, (**k**) λ_2_ = 4.35 μm, and (**l**) λ_3_ = 4.90 μm, respectively.

The nanostructured two graphene nanoribbon had a spatial period of *L* = 200 nm along *xy*-plane, which is sandwiched with the substrate and superstrate in Figure 1a. The refraction index of superstrate was set as *n*_1_. The FDTD method was used for the calculation. All components of electric and magnetic can be defined in the Yee’s grid. The perfectly matched absorbing boundary conditions were employed along the z direction, and the periodic boundary conditions were used along the x and y directions. Four-unit cells along the *xy* plane are plotted in Figure 1a; only one unit cell was considered in the computational space, which contained two graphene nanoribbons. It provided some guidance for the sample fragments with close spacing of each graphene nanoribbon because it was difficult to fabricate a perfect array with one graphene nanoribbon. The x direction polarized incident wave propagated along the *z*-axis. The grid size of 1 nm was used to mesh the graphene thin film. The graphene nanoribbon was modeled with cuboid (length *P*_1_, width *P*_2_, thickness Δ): *P*_1_ = 160 nm along *y* axis, *P*_2_ = 40 nm along *x* axis and Δ = 1 nm along *z* axis. The space between the two graphene nanoribbons was *d* = 40 nm. The material SiO_2_ was set as dielectric substrate. The conductivity had the form [40]:

σ_gra_(*ω*) = i*e*^2^**E***_f_/* [π*ħ*^2^(*ω* + i*τ*^−1^)]. (1)

The anisotropic dielectric tensor is given by *ε*_gra_ = (*ε*_11_, *ε*_22_, *ε*_33_). The two components of dielectric tensor in *xy*-plane are set with *ε*_11_ = *ε*_22_ = *ε*_0_ (1 + iσ_gra/_(*ε*_0_*ω*Δ)). The component with *ε*_33_ = *ε*_0_ was along the *z* direction. Here, the electric charge is *e*, and the reduced Planck’s constant is *ħ*. The vacuum permittivity with *ε*_0_, Fermi velocity with ν*_f_*, Fermi energy with **E***_f_*, carrier mobility with μ and carrier relaxation time with *τ* were used. The parameters about graphene nanoribbon were used here with **E***_f_* = 0.64eV, μ = 1 m^2^/(V·s), ν*_f_* = 10^6^ m/s and *τ* = (μ**E***_f_*)/(*e*ν*_f_* ^2^).

When the incident light S_1,in_ was coupled into the nanostructured GN grating, the characteristics of the GSP was analyzed using theoretical CMT. The field *a*_m_ (m = 1, 2, 3) of GSP modes has equation *da*_m_/*dt* = −i*ω*_m_*a*_m_, where the resonance frequency is *ω*_m_. The evolution for *a*_m_ can be described with the theoretical CMT model of the nanostructured GN grating [39,40]:

(2) $$\frac{\partial }{\partial t}|a\rangle =-j\Omega |a\rangle -\left(\Gamma_{i}+\Gamma_{w}\right)|a\rangle +S_{1,\mathrm{in}}|K\rangle +S_{2,\mathrm{in}}|K\rangle -M|a\rangle$$

(3) $$S_{2,\mathrm{out}}=-S_{2,\mathrm{in}}+\langle K|a\rangle$$

(4) $$S_{1,\mathrm{out}}=-S_{1,\mathrm{in}}+\langle K|a\rangle$$

(5) $$|a\rangle =\left(\begin{array}{c} a_{1}\\ \begin{array}{c} a_{2}\\ a_{3} \end{array} \end{array}\right),\;|K\rangle =\left(\begin{array}{c} k_{1}\\ \begin{array}{c} k_{2}\\ k_{3} \end{array} \end{array}\right),\langle K|=\left(k_{1}\;\;k_{2}\;\;k_{3}\right)$$

(6) $$\Omega =\left[\begin{array}{ccc} \omega_{11} & \omega_{12} & \omega_{13}\\ \omega_{21} & \omega_{22} & \omega_{23}\\ \omega_{31} & \omega_{32} & \omega_{33} \end{array}\right]\Gamma_{w}=\left[\begin{array}{ccc} \gamma_{w11} & \gamma_{w12} & \gamma_{w13}\\ \gamma_{w21} & \gamma_{w22} & \gamma_{w23}\\ \gamma_{w31} & \gamma_{w32} & \gamma_{w33} \end{array}\right],\Gamma_{i}=\;\left[\begin{array}{ccc} \gamma_{i11} & \gamma_{i12} & \gamma_{i13}\\ \gamma_{i21} & \gamma_{i22} & \gamma_{i23}\\ \gamma_{i31} & \gamma_{i32} & \gamma_{i33} \end{array}\right],M=\left[\begin{array}{ccc} \mu_{11} & \mu_{12} & \mu_{13}\\ \mu_{21} & \mu_{22} & \mu_{23}\\ \mu_{31} & \mu_{32} & \mu_{33} \end{array}\right]$$

1/Q_m_ = 1/Q_im_ + 1/Q_wm_. (7)

*τ*_m_ = Q_m_/*ω*_m_. (8)

where S_1,in_, S_2,in_, S_1,out_, and S_2,out_ represent the amplitude for incoming and outgoing waves, respectively. |a⟩ represents the amplitude of resonant GSP modes and |K⟩ is the coupling coefficient between GSP modes and light field. *k*_m_ stands for the coupling between each GSP mode and external light field. The Ω matrix represents resonant frequencies, the Γ_w_ matrix represents external loss rate, the Γ_i_ matrix represents the intrinsic loss rate, and the Μ matrix represents coupling coefficients, respectively. The m and n are set to 1, 2 and 3. If n ≠ m, *ω*_mn_, γ_wmn_, γ_imn_, Q_wmn_ and Q_imn_ are all equal to zero; if m = n, we have the relations *ω*_mn_ = *ω*_m_, γ_imn_ = γ_im_, γ_wmn_ = γ_wm_, Q_wmn_ = Q_wm_, Q_imn_ = Q_im_, μ_mn_ = 0, γ_wm_ = *ω*_m_/(2Q_wm_) = 1/(2*τ*_wm_), Q_wm_ = *ω*_m_*τ*_wm_, γ_im_ = *ω*_m_/(2Q_im_) =1/(2*τ*_im_) and Q_im_ = *ω*_m_*τ*_im_. The decay rate γ_im_ represents the intrinsic loss process for m-th GSP mode, and Q_im_ is the quality factor for the corresponding decay process of intrinsic loss. The decay rates γ_wm_ represent the energy coupling loss process between the m-th GSP mode and external light field; and Q_wm_ is the quality factor for this energy coupling loss process. The coupling coefficient is μ_mn_, which represents the coupling between three resonant GSP, which has the relation μ_mn_ = μ_nm_. The *τ*_wm_ and *τ*_im_ are the lifetime. The relations between the quality factor and lifetime for corresponding processes at the m-th GSP mode have the equations Q_wm_ = *ω*_m_*τ*_wm_ and Q_im_ = *ω*_m_*τ*_im_. For the GN plasmonic-photonic absorber structure, the total quality factor Q_m_, the total lifetime *τ*_m_ and *ω*_m_ have the relations in Equations (7) and (8).

With the initial condition S_2, in_ = 0, the reflection function, transmission function and absorption *A*(*ω*) with using the CMT method are obtained with:

(9) $$r(\omega )=-\left(\sqrt{\gamma_{w1}}G_{1}+\sqrt{\gamma_{w2}}G_{2}+\sqrt{\gamma_{w3}}G_{3}\right)/G_{0}$$

(10) $$t(\omega )=1-\left[\left(\sqrt{\gamma_{w1}}+\sqrt{\gamma_{i1}}\right)G_{1}-\left(\sqrt{\gamma_{w2}}+\sqrt{\gamma_{i2}}\right)G_{2}-\left(\sqrt{\gamma_{w3}}+\sqrt{\gamma_{i3}}\right)G_{3}\right]/G_{0}$$

(11) $$A\left(\omega \right)=1-{\left|t\left(\omega \right)\right|}^{2}-{\left|r\left(\omega \right)\right|}^{2}$$

where γ_m_ = −(*ω*_m_-*ω*)i − γ_wm_ − γ_im_. The χ_1_ = iμ_12_, χ_2_ = iμ_13_, χ_3_ = iμ_23_. *G*_0_, *G*_1_, *G*_2_ and *G*_3_ are the function of γ_m_, χ_1_, χ_2_ and χ_3_, where $G_{0}=2\chi_{1}\chi_{3}\chi_{2}+\chi_{1}\chi_{1}\gamma_{3}+\chi_{2}\chi_{2}\gamma_{2}+\chi_{3}\chi_{3}\gamma_{1}-\gamma_{1}\gamma_{2}\gamma_{3}$, $G_{1}=\left(\gamma_{2}\gamma_{3}-\chi_{3}\chi_{3}\right)\sqrt{\gamma_{w1}}+\left(\chi_{1}\gamma_{3}+\chi_{2}\chi_{3}\right)\sqrt{\gamma_{w2}}+\left(\chi_{2}\gamma_{2}+\chi_{1}\chi_{3}\right)\sqrt{\gamma_{w3}}$, $G_{2}=\left(\chi_{1}\gamma_{3}+\chi_{2}\chi_{3}\right)\sqrt{\gamma_{w1}}+\left(\gamma_{1}\gamma_{3}-\chi_{2}\chi_{2}\right)\sqrt{\gamma_{w2}}+\left(\gamma_{1}\chi_{3}+\chi_{2}\chi_{1}\right)\sqrt{\gamma_{w3}}$, and $G_{3}=\left(\chi_{2}\gamma_{2}+\chi_{3}\chi_{1}\right)\sqrt{\gamma_{w1}}+\left(\gamma_{1}\chi_{3}+\chi_{1}\chi_{2}\right)\sqrt{\gamma_{w2}}+\left(\gamma_{1}\gamma_{2}-\chi_{1}\chi_{1}\right)\sqrt{\gamma_{w3}}$. We can compare the absorption spectra *A*(*ω*) obtained by the CMT theory in Equation (11) with the absorption simulated by FDTD method. With using Q_wm_ = *ω*_m_/(2γ_wm_) and Q_im_ = *ω*_m_/(2γ_im_), we can get values such as *ω*_m_, Q_wm_, Q_im_, *τ*_wm_ and *τ*_im_, respectively. Moreover, the total quality factor Q_m_ and total lifetime *τ*_m_ for the m-th GSP mode can be calculated. 

## 3. Field Enhancement and Photon Flux Density of GSP

We concentrated on the properties and behavior of collections of photons, which is investigated by the nature of the GN surface plasmon wave. The absorptions with different width and lattice period using the FDTD simulation are shown here in Figure 1b,c. The absorption with different width *P*_2_ = 30 nm (black line), 40 nm (blue line), 50 nm (red line) and 60 nm (green line) of the GN grating was investigated, shown in Figure 1b. The second resonant mode (blue-dotted arrow) and third resonant mode (red-dotted arrow) both have the red-shift. The absorption with different lattice period *L* = 200 nm (black line), 300 nm (blue line) and 400 nm (red line) in Figure 1c, respectively. The first resonant mode (black-dotted arrow) and the third resonant mode (red-dotted arrow) are almost unchanged. The second resonant mode (blue-dotted arrow) has the blue-shift. For the case of the lattice period *L* = 200 nm in Figure 1c, three different GSP resonance modes have the resonant wavelengths λ_1_ = 3.9 μm, λ_2_ = 4.35 μm and λ_3_ = 4.91 μm, respectively. From the absorption calculation with FDTD method, we can obtain the resonant frequency value *ω*_m_ in Equation (11) with *ω*_m_ = 2πc/λ*_m_*. The absorption *A*(*ω*) can be fitted by choosing the values of γ_im_ and γ_wm_ in Equation (11). By comparing the intensity and spectral width of absorption *A*(*ω*) obtained by CMT in Equation (11) with that of absorption (case for *L* = 200 nm) simulated by FDTD in Figure 1c, we can get the values: (γ_w1_, γ_w2_, γ_w3_) = (2.2 × 10^9^ rad/s, 10.8 × 10^10^ rad/s, 12.2 × 10^10^ rad/s), and (γ_i1_, γ_i2_, γ_i3_) = (1.25 × 10^12^ rad/s, 1.83 × 10^12^ rad/s, 1.87 × 10^12^ rad/s). Using Q_wm_ = *ω*_m/_(2γ_wm_), Q_im_ = *ω*_m_/(2γ_im_), *τ*_wm_ = 1/(2γ_wm_), *τ*_im_ = 1/(2γ_im_), Q_w_ = *ω*_m_ *τ*_w_ and the relations in Equations (7) and (8), we can get quality factors: (Q_w1_, Q_w2_, Q_w3_) = (4.7 × 10^4^, 2.04 × 10^3^, 1.59 × 10^3^), (Q_i1_, Q_i2_, Q_i3_) = (192, 118, 103), (Q_1_, Q_2_, Q_3_) = (192, 112, 97) and lifetimes (*τ*_w1_, *τ*_w2_, *τ*_w3_) = (0.2 ns, 4.7 ps, 4.15 ps), (*τ*_i1_, *τ*_i2_, *τ*_i3_) = (397 fs, 275 fs, 270 fs) and (*τ*_1_, *τ*_2_, *τ*_3_) = (396 fs, 260 fs, 254 fs). The μ_mn_ is set as 1.0 × 10^4^ rad/s in this model.

It is represented that the distributions of the electric field component **E**_z_, electric field component **E**_x_, and photon flux density Φ at λ_1_ = 3.9 μm, λ_2_ = 4.35 μm and λ_3_ = 4.91 μm are obtained with FDTD simulation in Figure 1d–l, respectively. The distributions of the electric field component **E**_z_ at λ_1_, λ_2_, and λ_3_ are shown in Figure 1d–f, respectively. The distributions of the electric field component **E**_x_ at λ_1_, λ_2_, and λ_3_ are shown in Figure 1g–i, respectively. The distributions of the photon flux density Φ at λ_1_, λ_2_, and λ_3_ are shown in Figure 1j–l, respectively. The left and right edges corresponding to the polarization of the incident light have strong local photon flux density Φ at λ_1_ = 3.9 μm and λ_2_ = 4.35 μm, whose magnitude decays very fast outside the graphene nanoribbons edge in Figure 1j–k. The Φ at λ_2_ = 4.35 μm is also seen to be partly localized inside the graphene nanoribbons region due to the short-range interaction in Figure 1k. The Φ with λ_3_ = 4.91 μm is seen to be located at the four corners of the graphene nanoribbon, which is a corner effect as shown in Figure 1l. The photon flux density Φ at λ_3_ = 4.91 μm has reduced to almost zero inside the center region of graphene nanoribbons.

The amplitude of electric field |**E**(λ_m_)| for the m-th GSP mode had a function of **r**_0_, Fermi energy **E***_f_*, carrier mobility μ and refractive index *n*_1_, which can be calculated as |**E**(λ_m_, **r**_0_, **E***_f_*, μ, *n*_1_)| with FDTD simulation:

|**E**(λ_m_, **r**_0_, **E***_f_*, μ, *n*_1_)| = sqrt(**E**_x_^2^+ **E**_y_^2^+ **E**_z_^2^) (12)

where **r**_0_ is a position in the grapheme region. We concentrated on the property and behavior of collections of photons, which is determined by the nature of graphene surface plasmon wave. Monochromatic light of a frequency ω_m_ (m = 1, 2, 3) and intensity *I* (w/m^2^) carries a mean photon flux density Φ. The distribution of photon flux density Φ for the *m*-th GSP mode (m = 1, 2, 3) can be calculated as:

(13) $$\Phi =I(\lambda_{m},\;r_{0},\;E_{f},\;\mu ,n_{1})/h\omega m_{\;}=E(\lambda_{m},\;r_{0},\;E_{f},\;\mu ,n_{1})\;E*(\lambda_{m},\;r_{0},\;E_{f},\;\mu ,n_{1})/h\omega m$$

For tunable graphene nanoribbons plasmonic-photonic absorber, we can get the tunable ability of electric field |**E**(λ_m_)| and photon flux density Φ using Fermi energy **E***_f_*, carrier mobility μ and refractive index *n*_1_.

**Figure 2:**
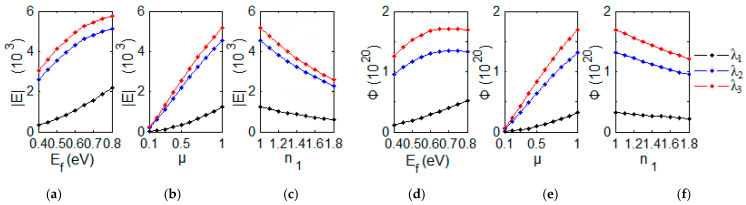
The amplitude of electric field (|E|) with different (**a**) **E***_f_*, (**b**) μ, (**c**) *n*_1_, and photon flux density Φ with different (**d**) **E***_f_*, (**e**) μ, (**f**) *n*_1_ at resonant wavelengths λ_1_, λ_2_ and λ_3_ inside the graphene region with FDTD simulation, respectively.

To get more insight into the field localization and corresponding photonic localization, electric field and photon flux density with different modulated parameters **E***_f_* are studied with FDTD simulation in Figure 2a–f. The parameters are fixed with μ = 1 m^2^/(V·s) and *n*_1_ = 1 for various Fermi level **E***_f_*. With the FDTD simulation, the amplitude of electric field |**E**(λ_m_)| and photon flux density Φ(λ_m_) (m = 1, 2, 3) at λ_1_, λ_2_ and λ_3_ inside the graphene region with various Fermi energy **E***_f_* are depicted in Figure 2a,d). For **E***_f_* = 0.4 eV, the three different resonant modes with wavelengths λ_1_ = 4.94 μm, λ_2_ = 5.51 μm and λ_3_ = 6.21 μm are obtained with the FDTD simulation. Then, we can obtain |**E**(λ_m_)| and Φ(λ_m_) for the case **E***_f_* = 0.4 eV in grapheme region using Equations (12) and (13). For other cases **E***_f_* = 0.45, 0.50, 0.55, 0.60, 0.65, 0.70, 0.75, and 0.80 eV, both |**E**(λ_m_)| and Φ(λ_m_) can also be obtained in grapheme region, respectively. The values of |**E**(λ_m_)|, Φ(λ_m_) with different Fermi level **E***_f_* have the fitting expresses as the follows: |**E**(λ_1_)| = 5.3**E***_f_*^2^ − 1.7**E***_f_* + 0.2 (10^3^), |**E**(λ_2_)| =−11.7**E***_f_*^2^ + 20.4**E***_f_* − 3.7 (10^3^), |**E**(λ_3_)|= −13.7**E***_f_*^2^ + 23.2**E***_f_* − 4.1 (10^3^), Φ(λ_1_) = 0.6**E***_f_*^2^ + 0.2**E***_f_* − 0.1 (10^19^), Φ(λ_2_) = −3.9**E***_f_*^2^ + 5.6**E***_f_* − 0.7 (10^20^) and Φ(λ_3_) = −5.0**E***_f_*^2^ + 7.1**E***_f_* − 0.8 (10^20^). For plasmonic-photonic absorber, we can get the tunable ability of electric field amplitude |**E**(λ_m_)| and photon flux density Φ using Fermi energy **E***_f_*, which can be adjusted with applied voltage bias or doping concentration.

If we set Fermi level **E***_f_* = 0.64 eV and *n*_1_ = 1, we can study similar field localization and corresponding photonic localization with different carrier mobility μ. The amplitude of electric field |**E**(λ_m_)| and photon flux density Φ(λ_m_) at λ_1_, λ_2_ and λ_3_ inside the graphene region with various carrier mobility μ are depicted in Figure 2b,e. The values of |**E**(λ_m_)|, Φ(λ_m_) with different carrier mobility μ have the fitting expresses: |**E**(λ_1_)|= 1.1μ^2^ + 0.2μ − 0.02 (10^3^), |**E**(λ_2_)|= 4.9μ − 0.3 (10^3^), |**E**(λ_3_)|= 5.6μ − 0.3 (10^3^), Φ(λ_1_) = 0.3μ^2^ + 0.1μ − 0.005 (10^19^), Φ(λ_2_) = 1.4μ − 0.1 (10^20^), and Φ(λ_3_) = 1.8μ − 0.1 (10^20^). The electric field |**E**(λ_m_)| and photon flux density Φ can be adjusted with the type and quantity of impurities, and working temperature. The corresponding sensors probe the impurity and analyzing temperature [41].

For fixed **E***_f_* = 0.64 eV and carrier mobility μ = 1 m^2^/(V·s), the field localization and corresponding photonic localization are sensitive with refractive index *n*_1_ of superstrate, which has sensing application in detecting the surrounding environment. The amplitude of electric field |**E**(λ_m_)| and Φ(λ_m_) at λ_1_, λ_2_ and λ_3_ inside the graphene region with various refractive index *n*_1_ are depicted in Figure 2c,f. The fitting expresses are set as follows: |**E**(λ_1_)|= −0.8*n*_1_ + 2.0 (10^3^), |**E**(λ_2_)|= −2.8*n*_1_ + 7.2 (10^3^), |**E**(λ_3_)|= −3.2*n*_1_ + 8.2 (10^3^), Φ(λ_1_) = −0.1*n*_1_ + 0.5 (10^19^), Φ(λ_2_) = −0.5*n*_1_ + 1.8 (10^20^) and Φ(λ_3_) = −0.6*n*_1_ + 2.3 (10^20^). The theoretical descriptions of the tunable photon flux density Φ will make it useful in applying the theory for sensing applications by changing the refractive index *n*_1_ of superstrate such as aqueous solution [39].

## 4. Tuning Quality Factor and Lifetime of GSP

The field localization and corresponding photonic localization can be adjusted with applied voltage bias, doping concentration, impurities, working temperature and refractive index *n*_1_ of superstrate. The quality (Q) factor of the system may vary greatly with different applications and requirements. The system, with an emphasis on damping, only needs a low-quality factor. The Q factor of atomic clock, accelerators, laser or other optical resonators, which need strong resonance or frequency stability, is high. And their Q factor can reach 10^11^ or even higher. Trapped light within the radiation continuum has been experimentally measured, and the ability to tune the maximal radiative Q from infinite to finite is an unique property that may be exploited [42]. High Q-factor indicates that the energy loss rate of the oscillator is slow, and the vibration lasts for a long time. It is necessary that the quality factor and lifetime of GSP in plasmonic-photonic devices can undergo tuning.

The energy exchange of plasmonic-photonic absorber has mainly two ways: one way is the decay process of intrinsic loss for each resonant mode and another is the decay process of coupling loss between the GSP mode and the external light field. Q_im_ and *τ*_im_ are the quality factor and lifetime of the first decay process. Q_wm_ and *τ*_wm_ are the quality factor and lifetime of the second decay process. For the plasmonic-photonic absorber, the total quality factor Q_m_ and total lifetime *τ*_m_ can be obtained with the relations in Equations (7) and (8).

**Figure 3:**
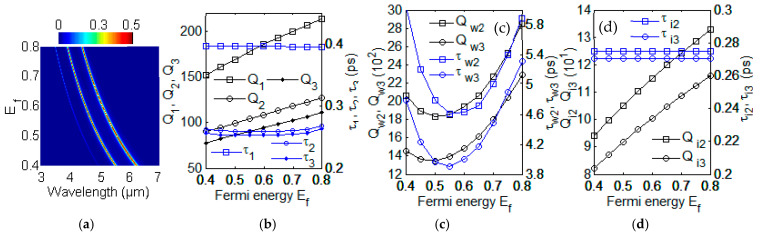
(**a**) The evolution of optical absorption spectra with different Fermi energy with using FDTD simulation. (**b**) Q_1_, Q_2_, Q_3_ and *τ*_1_, *τ*_2_, *τ*_3_ with different Fermi energy **E***_f_*. (**c**) Q_w2_, Q_w3_ and *τ*_w2_, *τ*_w3_ with different Fermi energy **E***_f_*. (**d**) Q_i2_, Q_i3_ and *τ*_i2_, *τ*_i3_ with different Fermi energy **E***_f_*.

The GSP resonances can be used for modulation using electric voltage bias. There was a good broad tunability of the GSP by changing the Fermi energy **E***_f_*. The parameter of carrier mobility is fixed with μ = 1 m^2^/(V·s) and *n*_1_ = 1 here. The photoresponsivity of GN grating strongly depends on the Fermi energy **E***_f_*. The evolution of optical absorption spectra for different Fermi energy **E***_f_* is investigated with FDTD simulation in Figure 3a. The three GSP modes have the blue shift with the increasing of Fermi energy **E***_f_*. For **E***_f_* = 0.40 eV, the graphene surface plasmon have three resonant wavelengths λ_1_ = 4.94 μm, λ_2_ = 5.51 μm and λ_3_ = 6.21 μm with the FDTD simulation. The absorption *A*(*ω*) in Equation (11) can be obtained with appropriate values of γ_im_ and γ_wm_. By comparing the intensity and spectral width of absorption *A*(*ω*) in Equation (11) with that of absorption simulated with FDTD in Figure 3a, we can get the fitting values: (γ_w1_, γ_w2_, γ_w3_) = (2.2 × 10^8^ rad/s, 8.2 × 10^10^ rad/s, 1.04 × 10^11^ rad/s) and (γ_i1_, γ_i2_, γ_i3_) = (1.26 × 10^12^ rad/s, 1.81 × 10^12^ rad/s, 1.85 × 10^12^ rad/s). Using Q_wm_ = *ω*_m_/(2γ_wm_), Q_im_ = *ω*_m_/(2γ_im_), *τ*_wm_ = 1/(2γ_wm_), *τ*_im_ = 1/(2γ_im_), Q_w_ = *ω*_m_*τ*_w_ and the relations in Equations (7) and (8), we can get quality factors: (Q_w1_, Q_w2_, Q_w3_) = (8.67 × 10^5^, 2.06 × 10^3^, 1.45 × 10^3^), (Q_i1_, Q_i2_, Q_i3_) = (151, 94, 82), (Q_1_, Q_2_, Q_3_) = (151, 90, 78) and lifetimes (*τ*_w1_, *τ*_w2_, *τ*_w3_) = (2.27 ns, 6.02 ps, 4.78 ps), (*τ*_i1_, *τ*_i2_, *τ*_i3_) = (397 fs, 275 fs, 270 fs), (*τ*_1_, *τ*_2_, *τ*_3_) = (397 fs, 263 fs, 256 fs). For other **E**_f_ = 0.45, 0.50, 0.55, 0.60, 0.65, 0.70, 0.75, and 0.80 eV, all values of total quality factor Q_m_ and total lifetime *τ*_m_ using CMT method are shown in Figure 3b. 

For graphene plasmonic-photonic structure at m-th GSP mode (m = 1, 2, 3), the quality factor Q*_m_* and lifetime *τ_m_* with different Fermi energy **E***_f_* are shown in Figure 3b_._ With the fixed value μ = 1 m^2^/(V·s) and *n*_1_ = 1, the Q*_m_* increase as the increasing of Fermi energy **E***_f_* in Figure 3b. The *τ*_m_ has little change as the **E***_f_* increasing from 0.40 eV to 0.70 eV. The *τ*_2_ and *τ*_3_ increase lightly with the change of Fermi energy **E***_f_* from 0.7 eV to 0.8 eV. According to the different applications and requirements, the total quality factor Q_m_ and lifetime *τ_m_* of three GSP modes can be modulated with **E***_f_* by changing voltage bias or doping concentration. Quality factors Q_w2_, Q_w3_ and lifetimes *τ*_w2_, *τ*_w3_ with different Fermi energy **E***_f_* using CMT method are shown in Figure 3c. The quality factors Q_i2_, Q_i3_ and lifetimes *τ*_i2_, *τ*_i3_ with different Fermi energy **E***_f_* using CMT method are shown in Figure 3d. The values are shown with: Q_w1_ = 1.4 × 10^9^ exp(−12**E***_f_*^1/2^), Q_w2_ = 6.1 × 10^3^ − 1.6 × 10^4^ **E***_f_* + 1.5 × 10^4^ **E***_f_*^2^, Q_w3_ = 4 × 10^3^ − 1.1 × 10^4^ **E***_f_* + 1.1 × 10^4^ **E***_f_*^2^, Q_i1_ = 91 + 156 **E***_f_*, Q_i2_ = 56 + 97 **E***_f_* and Q_i3_ = 49 + 85 **E***_f_*. The intrinsic loss and coupling loss are investigated with changing Fermi energy **E***_f_*, which provide the insight into understanding two decay processes. Therefore, the graphene grating is a promising candidate for interesting electrically-controlled graphene nanoplasmonic-photonic devices.

**Figure 4:**
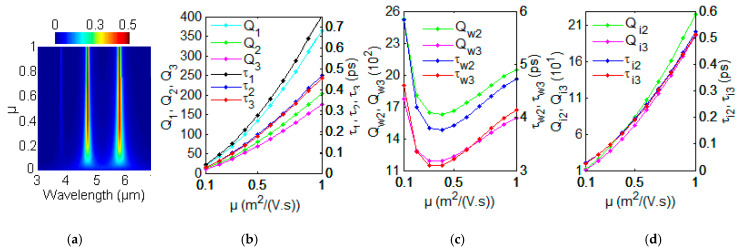
(**a**) The evolution of optical absorption spectra with different μ using FDTD simulation. (**b**) Q_1_, Q_2_, Q_3_ and *τ*_1_, *τ*_2_, *τ*_3_ with various μ_._ (**c**) Q_w2_, Q_w3_ and *τ*_w2_, *τ*_w3_ with various μ. (**d**) Q_i2_, Q_i3_ and *τ*_i2_, *τ*_i3_ with various μ.

The lower mobility in the graphene nanoribbon corresponds to higher loss with fixed Fermi energy **E***_f_* = 0.64 eV and *n*_1_ = 1. The evolution of optical absorption spectra for different carrier mobility μ is investigated with FDTD simulation as shown in Figure 4a. The resonant modes with λ_1_ = 3.9 μm, λ_2_ = 4.35 μm and λ_3_ = 4.91 μm keep unchanged with different carrier mobility μ. The absorption intensity of the three resonance modes in GSP system possesses the exponential decay with the decrease of carrier mobility μ. The lower mobility corresponds to a higher loss in the GN grating. The fitting parameters Q_wm_, Q_im_, γ_im_, γ_wm_, *τ*_im_ and *τ*_wm_ can be resented by comparing absorption *A*(*ω*) with absorption obtained by FDTD simulation. Then we can get Q_im_, Q_wm_,*τ*_wm_, *τ*_im_, Q_m_ and *τ*_m_ (m = 1, 2, 3) with various carrier mobility μ from μ = 0.1 to μ = 1 m^2^/(V·s). For graphene plasmonic-photonic structure at *m*-th GSP mode (m = 1, 2,3), the total quality factor Q_m_ and total lifetime *τ*_m_ are the function of carrier mobility μ obtained using CMT method in Figure 4b. In Figure 4b, the Q_m_ increase with the increase of μ. The total lifetime *τ*_m_ increase as the increasing of carrier mobility μ in Figure 4b. With the CMT method, the quality factors Q_w2_, Q_w3_ and lifetimes *τ*_w2_, *τ*_w3_ with different carrier mobility μ is shown in Figure 4c. With the CMT method, the quality factors Q_i2_, Q_i3_ and lifetimes *τ*_i2_, *τ*_i3_ with different carrier mobility μ is represented in Figure 4d. All the fitting values of parameters for the quality factors are obtained as follows: Q_w1_ = 7.7 × 10^9^ exp(−12μ^1/5^), Q_w2_ = 3.2 × 10^3^ − 8.9 × 10^3^μ + 1.5 × 10^4^μ^2^ − 7.5 × 10^3^μ^3^, Q_w3_ = 2.2 × 10^3^ − 6.1 × 10^3^μ + 1.1 × 10^4^μ^2^ − 5.2 × 10^3^μ^3^, Q_i1_= 2.3 × 10^−6^ + 172μ+ 191μ^2^, Q_i2_ = 3.4 × 10^−7^ + 107μ + 119μ^2^ and Q_i3_ = −1.5 × 10^−7^ + 94μ+ 104μ^2^. The theoretical descriptions and data fitting of decay rates or lifetimes will make it useful in to apply the methods for the change of carrier mobility μ in future modulated graphene devices. According to the different applications and requirements, the total quality factor Q_m_ and lifetime *τ*_m_ can be modulated by carrier mobility by changing the type and quantity of impurities and working temperature.

**Figure 5:**
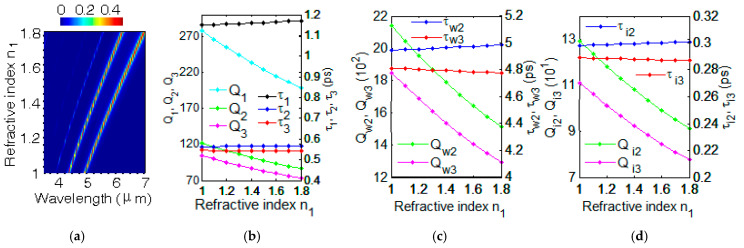
(**a**) The evolution of optical absorption spectra with different refractive index *n*_1_. (**b**) Q_m_ (m = 1, 2, 3) with various *n*_1_ of superstrate. (**c**) Q_w2_, Q_w3_ and *τ*_w2_, *τ*_w3_ with various n_1_. (**d**) Q_i2_, Q_i3_ and *τ*_i2_, *τ_i_*_3_ with various *n*_1_.

Here, we fix the values of **E***_f_* = 0.64 eV and μ = 1 m^2^/(V·s). The evolution of simulated optical absorption spectra for different refractive index *n*_1_ is investigated with the FDTD method, as shown in Figure 5a. The fitting values of Q_wm_, Q_im_, *τ*_wm_, *τ*_im_, γ_wm_ and γ_im_ can be represented after comparing absorption *A*(*ω*) in Equation (11) with that obtained by FDTD simulation in Figure 5a. The three resonance GSP modes have the red-shift with increasing of refractive index *n*_1_ in Figure 5a. This quasi-linear response characteristic between the *n*_1_ and resonant wavelength is especially valuable for the sensing application of graphene. For the graphene plasmonic-photonic structure at the m-th GSP mode (m = 1, 2, 3), the total quality factor Q_m_ and total lifetime *τ*_m_ are shown as the function of the refractive index *n*_1_ of superstrate using CMT method in Figure 5b. In Figure 5b, the total quality factors Q_m_ decreased as the refractive index *n*_1_ increased from 1 to 1.8, while the total lifetime *τ*_m_ remained nearly unchanging with the various refractive index *n*_1_. With the CMT method, the quality factors Q_w2_, Q_w3_ and lifetimes *τ*_w2_, *τ*_w3_ with different refractive index *n*_1_ is shown in Figure 5c. With the CMT method, the quality factors Q_i2_, Q_i3_ and lifetimes *τ*_i2_ and *τ*_i3_ with different refractive index *n*_1_ are shown in Figure 5d. All the values of quality factors are represented as follows: Q_w1_ = − 22,375 *n*_1_ + 1.1726 × 10^5^, Q_w2_ = − 784 *n*_1_ + 2902, Q_w3_ = −696 *n*_1_ + 2526, Q_i1_ = 10 *n*_1_ + 38, Q_i2_ = −5 *n*_1_ + 17, Q_i3_ = 4 *n*_1_ + 15. The theoretical descriptions and data fitting of quality factors and lifetimes with different *n*_1_ of superstrate, such as aqueous solution, will make it useful to apply the methods for future 2D materials modulation and sensing devices.

How to describe the Figure of merit (FOM) of a sensor? It can be related to the resonance wavelength shifts at certain refractive index *n*_1_. The sensitivity can be defined as [38,39]. Resonant wavelength variation Δλ can be changed by the refractive index change Δ*n* of superstrate environment.

The carrier dynamic of a saturable structure plays an important role to determine how a short pulse can be produced in large-scale vertical bilayer junctions [23]. Let us assume that the incident field is ultrashort pulse here [43]. The short pulse with two different central frequencies *ω*_2_ and *ω*_3_ can be written as **E**(**r**,t) = **E**(**r**)exp(−(t − t_0_)^2^/(t_d_^2^)) (*e*^−i^*^ω^*^2^^t^ + *e*^−i^*^ω^*^3^^t^), where delay of time is t_0_ and t_d_ is 200 fs.

Oscillations and plasmon energy shift are studied in gold nanorods [44]. Experiment of the carrier dynamics in perovskite was observed [45,46]. The transient optical responses and dynamic evolution of the carrier in the 2D materials junction samples can be characterized using femtosecond differential transmission spectroscopy [19]. For thorough investigation of total energy *W* and lifetime *τ*_m_ for m-th resonant GSP mode, the transient dynamic evolution of optical responses can be investigated here using the FDTD code. The evolution of total energy *W* and total lifetime *τ*_m_ can be investigated with using the pulse excitation. The relation between the lifetime *τ*_m_ of the m-th resonant mode is the equations *τ*_m_ = *W*/*P*. The *W* is the total energy stored inside the computational region while *P* is the power radiation out from the grapheme region. *ω*_m_ represents frequency of the resonant GSP modes. The manipulation of the quality factor and lifetime of the GSP makes graphene an excellent application platform of the photoelectric/photothermal energy conversion process and higher harmonic generation.

**Figure 6:**
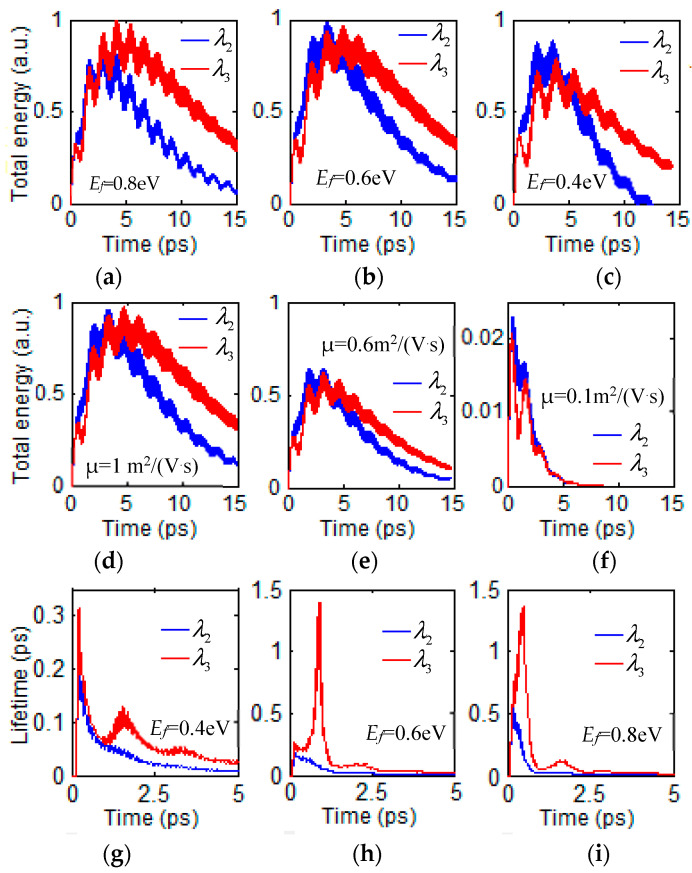
(**a**–**c**) The time evolution of total energy with time at λ_2_ and λ_3_ with **E***_f_* = 0.8 eV, 0.6 eV and 0.4 eV. (**d**–**f**) Evolution of total energy with time at λ_2_ and λ_3_ with μ = 1, 0.6 and 0.1 m^2^/(V·s). (**g**–**i**) The time evolution of lifetime at wavelengths λ_2_ and λ_3_ with various **E***_f_* = 0.4 eV, 0.6 eV and 0.8 eV.

First, we investigate the total energy *W* for m-th resonant GSP mode, which is stored inside the computational region. For the fixed value with μ = 1 m^2^/(V·s) and *n*_1_ = 1, the transient dynamic evolution of total energy at wavelengths λ_2_ and λ_3_ various with three different Fermi energy **E***_f_* from 0.8 eV to 0.4 eV obtained with FDTD simulation are plotted in Figure 6a–c, respectively. The total energy for **E***_f_* = 0.40 eV has a more rapidly decay than that of **E***_f_* = 0.60 eV and **E***_f_* = 0.80 eV. Moreover, the total energy of the mode λ_2_ has a more rapidly exponential decay than that of λ_3_. For plasmonic-photonic absorber, we can study the time evolution of total energy and its photon flux density by changing applied voltage bias. For the fixed value of **E***_f_* = 0.6 eV and *n*_1_ = 1, the time evolution of total energy at λ_2_ and λ_3_ with three different carrier mobility μ = 0.1 eV, 0.6 eV and 1 m^2^/(V·s) are represented with FDTD simulation in Figure 6d–f, respectively. The total energy has a more rapid decay when carrier mobility μ decreased from μ = 1 m^2^/(V·s) to μ = 0.1 m^2^/(V·s). The time evolution of total energy with different carrier mobility is helpful to probe the impurity and analyzing temperature. 

For the case μ = 1 m^2^/(V·s) and *n*_1_ = 1, the dynamics evolution of lifetimes at wavelengths λ_2_ and λ_3_ various with three different values of **E***_f_* = 0.4 eV, 0.6 eV and 0.8 eV are represented with FDTD simulation in Figure 6g–i, respectively. The lifetimes can almost reach 1.4 ps for the cases **E***_f_* = 0.6 eV and 0.80 eV. The lifetime of the mode λ_2_ has a more rapid exponential decay than that of λ_3_. For trapped light within the radiation continuum, the ability to tune the maximal radiative Q from infinite to finite is a unique property that may be exploited [42].

For the ultrafast dynamics of excited carriers in graphene, the time, energy, and momentum-resolved statistical distribution of hot electrons in quasi-free-standing graphene was directly measured after a photoexcitation process, which plays a central role for many electronic and optoelectronic applications [31]. The photoinduced carrier multiplication and carrier density were obtained from the electronic temperature. To study the dynamic evolution of the SP-induced hot carrier in the heterostructure of gold–graphene [44], the time-resolved differential reflection measurements were performed. After photoexcitation, the strongly out-of-equilibrium photocarriers rapidly thermalize distribution. The dominant mechanism for SP induced hot electron generation in the graphene originates from the near-field enhancement of direct photoexcitation in the graphene.

## 5. Second-Order and Third-Order Nonlinearity of GSP

**Figure 7:**
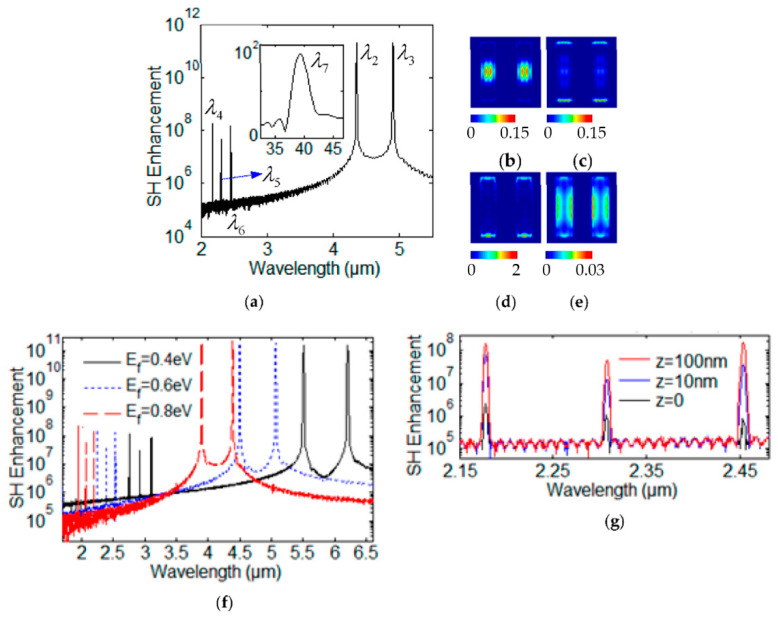
(**a**) Log plot of the spectrum of SH response. The distributions of Φ with different wavelengths (**b**) λ_4_, (**c**) λ_5_, (**d**) λ_6_, (**e**) λ_7_. (**f**) Log plot of the SH enhancement factor with three different Fermi energy **E***_f_* = 0.4 eV, 0.6 eV and 0.8 eV, respectively. (**g**) Log plot of the SH enhancement factor with three different position z = 0, z = 10 nm and z = 100 nm away from the graphene layer, respectively.

**Figure 8:**
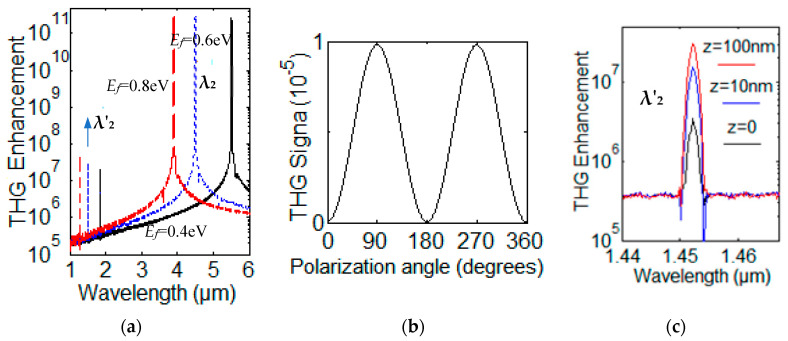
(**a**) Log plot of the THG enhancement factor with three different **E***_f_* = 0.40 eV (black solid line), 0.60 eV (blue dotted line) and 0.80 eV (red dash line) for FW modes λ_2_. (**b**) Polar diagram of polarization state of the THG emission at λ′_2_. (**c**) Log plot of the THG enhancement factor at λ′_2_ with three different position z = 0, z = 10 nm and z = 100 nm away from the graphene layer, respectively. The polarization state of the THG emission at λ_8_ is a function of the angle (not the polarization of the incident field θ = 0) corresponding to the x direction.

The high-efficiency second and third harmonic generation effects have been experimentally investigated in monolayer graphene-based transistors and exfoliated BP [21,22]. It was found that the local SP can also enhance the second and third harmonic generation (THG). The strongly localized fundamental field induces a desired increase of second harmonic wave and third harmonic wave. Here, we investigated the TH wave and SH wave including SHG as well as the SFG and DFG signals, whose results re calculated by the FDTD simulation in Figure 7 and Figure 8.

The incident electromagnetic field is composed of two monochromatic plane waves with frequencies *ω*_1_ and *ω*_2_: **E**(**r**,t) = **E**_2_*e*^i(*k*2·^**^r^**^−*ω*^^2t)^ + **E**_3_*e* ^i(*k*3·^**^r^**^−*ω*^^3t)^. Here, *k*_i_ (I = 2, 3) is the corresponding wave vector. Using the express of **E**(**r**,t), we can obtain the polarization of second-order nonlinear **P**^(2)^(**r**,t):

**P**^(2)^ = *ε*_0_χ^(2)^: [**E**_2_**E**_2_*e*
^2i(*k*2^^·^**^r^**^−*ω*^^2t)^ + **E**_3_**E**_3_*e*
^2i(*k*3^^·^**^r^**^−*ω*^^3t)^ + 2**E**_2_**E**_3_*e*
^i[(*k*2+*k*3)^^·^**^r^**^−(*ω*^^2^^+*ω*^^3)t]^ + 2**E**_2_**E**_3_*e
^i[(*k*2−*k*3)^^·^**^r^**^−^^(*ω*^^2−*ω*^^3)t]^ + cc]+2*ε*_0_χ^(2)^: (**E**_2_**E**_2_* + **E**_3_**E**_3_*) (14)

The terms in the Equation (15) have the second harmonic generation (SHG) signal at frequencies 2*ω*_2_ and 2*ω*_3_, difference frequency generation (DFG) signal with (*ω*_3_ − *ω*_2_) and sum frequency generation (SFG) signal with (*ω*_2_ + *ω*_3_), respectively. The value of χ^(2)^ can be obtained from Equation (15). The Equation (15) represents the nonlinear optical processes, including SHG, SFG, and DFG. When the fundamental wave (FW) light with two frequencies is incident upon the GN grating, SHW will be excited. It is noted that observation of optical second harmonic generation from suspended single-layer and bi-layer grapheme was experimentally reported [21].

Let us now assume that the incident electromagnetic field is the superposition of two monochromatic plane waves. These incident electric fields with two frequencies *ω*_2_ and *ω*_3_ can be written as: **E**(**r**,t) = **E**_0_(**r**)(*e*^−i^*^ω^*^2t^ + *e*^−i^*^ω^*^3t^). For incident wave with λ_2_ = 4.35 μm and λ_3_ = 4.91 μm, the Fourier spectrum of **E**_x_ propagating away from structure is shown in Figure 7a. There are four GSP modes for second-order nonlinear spectrum with four resonant wavelengths λ_4_ = 2.17 μm, λ_5_ = 2.31 μm, λ_6_ = 2.45 μm, and λ_7_ = 38.87 μm in Figure 7a. The SHG modes with λ_4_ = 2.17 μm and λ_6_ = 2.45 μm are resulted from the FW with wavelengths λ_2_ = 4.35 μm and λ_3_ = 4.91 μm due to the SHG effect, respectively. The SFG field has the wavelength λ_5_ = 2.31 μm. The resonant wavelength is λ_7_ = 38.87 μm for DFG field. The distributions of SH photon flux density Φ for λ_4_ = 2.17 μm, λ_5_ = 2.31 μm, λ_6_ = 2.45 μm, and λ_7_ = 38.87 μm are represented in Figure 7b–e, respectively. The distribution of SH photon flux density Φ at wavelength λ_4_ is seen to be mainly localized inside the graphene nanoribbons center region due to the short-range interaction in Figure 7b. The distribution of SH wave photon flux density Φ at wavelength λ_6_ is seen to be mainly localized along the short edge region in Figure 7d. The distribution of SH wave photon flux density Φ at wavelength λ_7_ is seen to be mainly localized along the four-edge region in Figure 7e.

Log plots of the SH enhancement factor with three different Fermi energy **E***_f_* = 0.40 eV, 0.60 eV and 0.80 eV are obtained in Figure 7f. Log plot of the SH enhancement factor with three different position z = 0, z = 10 nm and z = 100 nm away from the graphene layer with Fermi energy **E***_f_* = 0.64 eV are plotted in Figure 7g, respectively.

The third order nonlinear optical property in nonlinear 2D material graphene plasmonic-photonic absorber is investigated here. The Kerr effect of the third-order nonlinear polarization of graphene is expressed as following:

**P**^(3)^(*ω*) = 3*ε*_0_χ^(3)^|**E**(*ω*)|^2^**E**(*ω*). (15)

In this equation, third order susceptibility χ^(3)^ of graphene can be obtained in nonlinear 2D material. The graphene lattice with *D*_6h_ space group is centrosymmetric. A direct implication of this property is that second-order nonlinearity is forbidden. However, nonlinearity for TH wave is allowed and particularly strong in graphene. The quadratic optical nonlinearity of graphene can be described with using the nonlinear optical conductivity tensor σ_3_. The current density of third order nonlinear is **j**^3nl^(**r**,t) = σ_3_**E**(**r**,t)|**E**(**r**,t)|^2^. The nonlinear conductivity has the form [47]:

σ_3_(*ω*) = i*e*^2^**E***_f_*/π*ħ*^2^(*ω* + i*τ*^1^)] + 3i*e*^2^(*e*ν*_f_*)^2^(1 + α)/(32π*ħ*^2^**E***_f_*/*ω*^3^). (16)

Here, the imaginary of σ_3_ is negative, which describes the self-focusing type nonlinear response in graphene. Both linear conductivity σ_gra_ in Equation (1) and nonlinear conductivity σ_3_ in Equation (17) nonlinear conductivity are highly dependent on fermi energy, which could provide a way to get an electrically controlled optical biostability. After considering nonlinear effect, total conductivity has the form:

σ = σ_gra_ + σ_3_|**E**(**r**,t)|^2^. (17)

When the FW wave with one frequency is incident upon the GN grating, third-order nonlinearity will be excited. Log plot of the TH enhancement factor with three different Fermi energy **E***_f_* = 0.40 eV, 0.60 eV and 0.80 eV are shown for mode λ_2_ in Figure 8a. TH enhancement factor is about 10^−4^. The THG mode with wavelength λ′_2_ (blue-dotted line) was excited by the FW wave with wavelength λ_2_ (blue-dotted line). Illuminated with an x-polarized plane wave at the fundamental frequency λ_2_, the polarization state of the SH emission for amplitude of electric field (|E|) at λ′_2_ from an array of two graphene nanoribbons is shown in Figure 8b. The TH signal is a function of the angle (not the polarization of the incident field); the θ = 0 corresponds to the x axis. The log plot of the TH enhancement factor at TH mode λ_8_ with three different position z = 0, z = 10 nm and z = 100 nm away from the graphene layer is shown in Figure 8c. Recently, quantum confinement-induced enhanced third-order nonlinearity and carrier lifetime modulation in two-dimensional tin sulfide were observed with Z-scan measurements and fs-resolved transient absorption spectroscopy [48].

## 6. Conclusions

We investigate a framework of the local field enhancement, photon flux density, quality factor and lifetime for tunable graphene plasmonic-photonic structure and study the second order and third order nonlinear optical response of grapheme surface plasmons. The quality factor and lifetime for each process of intrinsic loss or coupling loss have been studied. We have investigated the modulated plasmonic-photonic absorber in two graphene nanoribbons grating using Fermi energy, carrier mobility and refractive index. The theoretical descriptions and data fitting will make it useful to apply the methods for future 2D material plasmonic-photonic structures, modulation, and devices application. The modulated 2D plasmonic-photonic absorber results from the enhanced local field. The strongly-localized fundamental field induces a desired increase of TH wave and SH wave, including SHG, as well as the SFG and DFG signals. The proposed configuration and results could provide the guidance for designing quality factor and lifetime modulated 2D material plasmonic-photonic devices and the active control of the photoelectric/photothermal energy conversion process such as solar energy conversion, nanoantenna, higher harmonic generation and high-sensitivity sensing.
